# Phase Attention Model for Prediction of Early Recurrence of Hepatocellular Carcinoma With Multi-Phase CT Images and Clinical Data

**DOI:** 10.3389/fradi.2022.856460

**Published:** 2022-03-24

**Authors:** Weibin Wang, Fang Wang, Qingqing Chen, Shuyi Ouyang, Yutaro Iwamoto, Xianhua Han, Lanfen Lin, Hongjie Hu, Ruofeng Tong, Yen-Wei Chen

**Affiliations:** ^1^Graduate School of Information Science and Engineering, Ritsumeikan University, Kusatsu, Japan; ^2^Department of Radiology, Sir Run Run Shaw Hospital, Zhejiang University, Hangzhou, China; ^3^College of Computer Science and Technology, Zhejiang University, Hangzhou, China; ^4^Graduate School of Information Science and Engineering, Yamaguchi University, Yamaguchi-shi, Japan; ^5^Zhejiang Lab, Research Center for Healthcare Data Science, Hangzhou, China

**Keywords:** early recurrence, deep learning, multi-phase CT images, intra-phase attention, inter-phase attention

## Abstract

Hepatocellular carcinoma (HCC) is a primary liver cancer that produces a high mortality rate. It is one of the most common malignancies worldwide, especially in Asia, Africa, and southern Europe. Although surgical resection is an effective treatment, patients with HCC are at risk of recurrence after surgery. Preoperative early recurrence prediction for patients with liver cancer can help physicians develop treatment plans and will enable physicians to guide patients in postoperative follow-up. However, the conventional clinical data based methods ignore the imaging information of patients. Certain studies have used radiomic models for early recurrence prediction in HCC patients with good results, and the medical images of patients have been shown to be effective in predicting the recurrence of HCC. In recent years, deep learning models have demonstrated the potential to outperform the radiomics-based models. In this paper, we propose a prediction model based on deep learning that contains intra-phase attention and inter-phase attention. Intra-phase attention focuses on important information of different channels and space in the same phase, whereas inter-phase attention focuses on important information between different phases. We also propose a fusion model to combine the image features with clinical data. Our experiment results prove that our fusion model has superior performance over the models that use clinical data only or the CT image only. Our model achieved a prediction accuracy of 81.2%, and the area under the curve was 0.869.

## Introduction

Hepatocellular carcinoma (HCC) is a primary liver cancer with a high mortality rate. It is one of the most common malignancies worldwide, especially in Asia, Africa, and southern Europe (1, 2). The main treatment options for HCC include surgical resection, liver transplantation, transarterial chemoembolization, targeted therapy, immunotherapy, and radiofrequency ablation. Doctors usually need to develop a proper and reasonable treatment approach based on the patient's lesion stage, physical condition, and wishes. For patients with well-preserved liver function, surgical resection is the first-line treatment strategy (3). Surgical resection is also the most common treatment (4). Patients have the longest survival period if the surgery is completed in one stage. However, the recurrence rate of HCC can reach 70–80% after surgical resection (5). HCC recurrence is also an important cause of patient death (6). Time to recurrence is an independent survival factor, and patients with early recurrence tend to have lower overall survival (OS) than patients with late recurrence (7, 8). It is important to identify patients at high risk of early recurrence of HCC after radical surgical resection.

To date, many studies have been performed to evaluate the prognosis of HCC patients after resection. Previous studies have shown that pathologic features, such as microvascular invasion (MVI), vascular tumor thrombosis, histologic grading, and tumor size, are factors in the prognostic risk stratification of HCC (9–11). However, the pathologic features can only be obtained by preoperative biopsy and cannot be widely used in routine clinical practice because of their aggressive nature and the risk of bleeding. Therefore, we need to develop a method to accurately predict the risk of early recurrence after resection prior to surgery.

Traditional approaches use machine learning methods (e.g., random forests or support vector machines) to construct predictive models based on patients' clinical data (12, 13). These methods ignore the medical imaging information of patients. Medical imaging is an integral part of the routine management of HCC patients and has become an important non-invasive tool for detecting and identifying the degree of malignancy of HCC (14, 15). However, conventional images obtain limited imaging features that do not fully reflect the heterogeneity within the tumor and are subjective assessments of the lesion made by physicians; the assessments show a high degree of variability among physicians. The use of such qualitative imaging features to accurately predict early recurrence in HCC patients remains challenging for physicians. In 2012, Lambin introduced the concept of radiomics, which uses machine learning techniques to extract many features from medical images to analyze disease and prognosis (16). Machine learning, on the other hand, is defined as a subclass of artificial intelligence systems and belongs to weak AI, which helps machines to learn and make decisions based on data (17). In 2015, Gillies et al. illustrated the effectiveness of radiomics, a quantitative method for extracting features from medical images (18). Since then, several studies have shown that the application of extracted medical image features can be used as prognostic imaging biomarkers (19, 20). Zhou et al. extracted 300 radiomic features from multi-phase computed tomography (CT) and screened 21 radiomic features to predict early recurrence of HCC using the least absolute shrinkage and selection operator (LASSO) regression method (21). Ning et al. also developed a CT-based radiomic model to predict early recurrence of HCC (22); they found that the integration of radiomic features and relevant clinical data could effectively improve the performance of the prediction model. Radiomics is a new tool for radiologists to provide quantitative analysis and image interpretation and to provide an automated process to remove repetitive tasks to save physician time and effort, improve diagnostic performance and optimize overall workflow (23). In addition, radiomics can facilitate a personalized approach to medicine by providing physicians with a non-invasive tool to change the way cancer patients are treated, which can allow patient-specific treatments (24, 25). Nowadays, problems such as the lack of standardization and proper validation of radiomics models hinder the practical application of radiomics-based technologies to clinical practice, and the establishment of large-scale image biobanks may be one way to solve the problem (26). In addition to this, deep learning-based radiomics models are considered as black boxes by clinicians, making these models less interpretable and practical, and these challenges will be further explored in future studies. However, the image features extracted in these two studies were based on handcrafted low- or mid-level image features, which are limited by a comprehensive description of the potential information associated with early recurrence. Manual tuning of the models also brings in human bias.

In recent years, deep learning has been applied to survival prediction for various cancers (27–29). Deep learning uses convolutional neural networks (CNNs) that can directly perform feature extraction and feature analysis on image inputs; deep learning uses an end-to-end network structure. End-to-end deep learning models can automatically extract relevant features from images without human intervention. Such models can eliminate human bias and can extract high-level semantic features that are limited by manually defined feature extraction (30). Although the predictive performance of deep learning has been shown to outperform radiomics approaches in other topics, a few studies have applied deep learning to early recurrence prediction in HCC. Yamashita et al. constructed a deep learning model to predict the recurrence of HCC based on digital histopathologic images with good results (31). However, digital histopathology images are usually obtained from resected tumors only after surgery, and they are difficult to apply to preoperative prediction.

Previous research has shown that deep learning methods make better predictions than radiomics methods (32, 33). We combined the patient's preoperative multi-phase CT images after registration into a single three-channel image as the input to the deep learning network. Although previous studies have yielded good results, there are still enhancements to be made. We can combine the correlation information between the three phases to further extract more important and critical features from the features extracted by CNN. The attention mechanism, which has become very popular recently, is applied to deep learning networks to obtain a breakthrough in the accuracy of many tasks in computer vision (34–36). In deep learning, the features extracted by the backward network flow are equally important. The attention mechanism can suppress the flow of some invalid information based on some a priori information, thus allowing important features to be retained. In this paper, we propose a prediction model based on deep learning, which contains intra-phase attention and inter-phase attention modules. The intra-phase attention module focuses on important information in different channels and spaces within the same phase, whereas the inter-phase attention module focuses on important information in different phases. We also propose a fusion model to combine the image features and clinical data.

## Materials

This study was approved by Zhejiang University, Ritsumeikan University, and Run Run Shaw Hospital. The medical images and clinical data used in this study were collected from Run Run Shaw Hospital. Initially, 331 consecutive HCC patients who underwent hepatectomy from 2012 to 2016 were included in this retrospective study. The following criteria were followed to select the patients: (1) patients with postoperatively confirmed HCC; (2) patients having a contrast-enhanced CT scan within a month prior to surgery; (3) patients undergoing postoperative follow-up for at least 1 year; (4) patients without any history of preoperative HCC treatment; and (5) patients with negative surgical margins (complete tumor resection). Ultimately, a cumulative total of 167 HCC patients (140 men and 27 women) were included in the study. The peak time of HCC recurrence was 1 year after resection, which was defined as “early recurrence” (ER) (37). Sixty-five (i.e., 38.9%) patients were identified as having early recurrence, whereas the remaining 102 (i.e., 61.1%) patients did not have any recurrence, that is, they were non-ER (NER). Therefore, these patients were divided into two groups: ER and NER.

### Clinical Data

Many studies have discussed clinical prognostic indicators of HCC recurrence. For example, Portolani et al. showed that chronic active hepatitis, such as the hepatitis C virus (HCV) infection, and tumor MVI were associated with ER (5). Examination of MVI is obtained by observing pathological sections after surgery. These clinical data were not included in our study. Chang et al. suggested a patient age of 60 years as the cut-off value for ER and NER (38). Okamura et al. (39) found that preoperative neutral lymphatic ratio (N/L ratio), an index of inflammation, was associated with disease-free survival and OS in HCC patients. Patients with NLR ≥ 2.81 had significantly better outcomes in the NLR <2.81 group as compared to those with NLR ≥ 2.81. In addition to NLR, general clinical indicators of prognosis included age, gender, tumor size, tumor number, hepatitis B virus (HBV) infection, portal vein invasion, alanine aminotransferase (ALT), alkaline phosphatase (AKP), glutamate transaminase (AST), Barcelona clinical liver cancer (BCLC) stage, cirrhosis, and alpha-fetoprotein (AFP) (40).

The clinical factors collected in our study are shown in Table 1 and include gender (male or female), age (<60 or ≥60 years), tumor size (<5 or ≥5 cm), number of tumors (single or multiple); portal vein NLR (<2.81 or ≥2.81), invasion (yes/no), CP level (A or B), cirrhosis (yes/no), HBV infection (yes/no), AST (<50 or ≥50 U/L), ALT (<40 or ≥40 U/L), and AKP (<125 or ≥125 U/L), BCLC staging (0, A, B, C), ALB (≥40 or <40 U/L), AFP (<9 or ≥9 μg/L), TB (<20.5 or ≥20.5 U/L), and GGT (<45 or ≥45 U/L). Clinical data were evaluated by the chi-squared test, a well-known method used to estimate dependencies between categorical variables (41, 42); *p*-values <0.05 were considered to be significant. Seven clinical factors, namely, tumor size, portal vein invasion, N/L ratio, TB, AFP, and BCLC stage, were selected and further expressed as a binary vector. The nine elements of the binary vector are [c1, c2, c3,..., c8, c9], where [c1] represents the age ([0]: <60, [1]: ≥60); [c2] represents the tumor size ([0]: <5 cm, [1]: ≥5 cm); [c3] represents the portal vein infiltration ([0]: absent, [1]: present); [c4] represents the N/L ratio ([0]: <2. 8, [1]: ≥2.8); [c5] represents TB ([0]: <20.5, [1]: ≥20.5); [c6] represents AFP ([0]: <9, [1]:≥ 9); [c7, c8, c9] represents the BCLC staging ([0, 0, 0]: 0, [0, 0, 1]: A, [0, 1, 0]: B, [1, 0, 0]: C).

**Table 1:** Clinical variables of patients with ER and NER.

**Clinical variables**	**Total** ***n* = 167**	**NER** ***n* = 102**	**ER** ***n* = 65**	*P*-value^*^ (Chi-square test)
Sex				0.826
Female	27	17	10	
Male	140	85	55	
Age				**0.018**
<60	102	55	47	
≥60	65	47	18	
Tumor size (mm)				**<0.001**
<50	102	74	28	
≥50	65	28	37	
Tumor number				0.152
Single	155	97	58	
Multiple	12	5	7	
Portal vein invasion				**0.001**
No	143	95	48	
Yes	24	7	17	
Liver cirrhosis				0.794
No	52	31	21	
Yes	115	71	44	
HBV infection				0.054
No	33	25	8	
Yes	134	77	57	
N/L ratio				**0.039**
<2.8	106	71	35	
≥2.8	61	31	30	
ALT (U/L)				0.417
<40	104	66	38	
≥40	63	36	27	
AST (U/L)				0.500
<50	123	77	46	
≥50	44	25	19	
AKP (U/L)				0.054
<125	131	85	46	
≥125	36	17	19	
GGT (U/L)				0.065
<45	66	46	20	
≥45	101	56	45	
ALB (g/L)				0.256
≥40	165	100	65	
<40	2	2	0	
TB (μmol/L)				**0.020**
<20.5	122	81	41	
≥20.5	45	21	24	
AFP (μg/L)				**0.003**
<9	50	39	11	
≥9	117	63	54	
CP level				0.132
A	140	89	51	
B	27	13	14	
BCLC stage				**<0.001**
0	22	17	5	
A	117	78	39	
B	4	1	3	
C	24	6	18	

*P <0.05 means significant difference. The bold values indicate P values <0.05 are highlighted. The bold values indicate the best results.

### Contrast-Enhanced CT Scan

**Figure 1:**
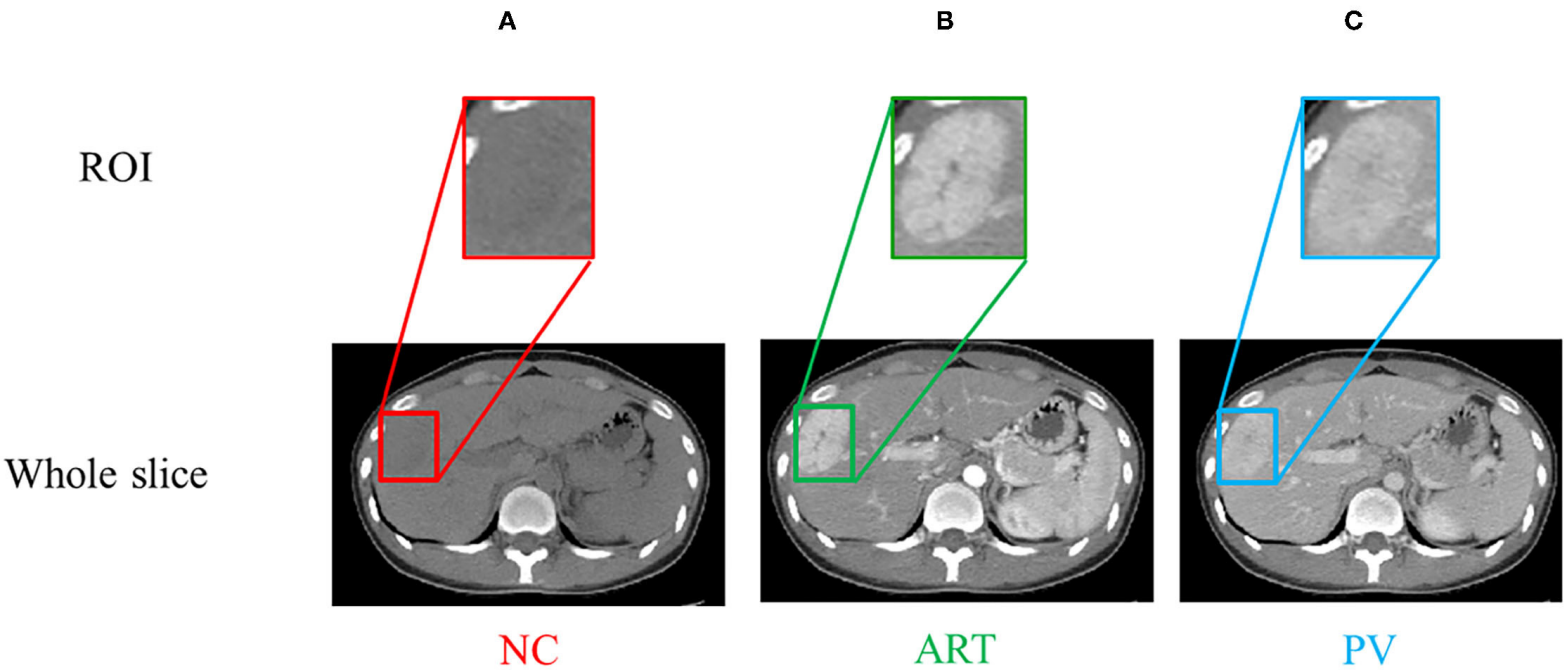
**(A–C)** are NC, ART, PV phases, respectively. The region of interest (ROI) is the bounding box of the tumor.

Contrast-enhanced CT scans (multi-phase CT images) are used for prediction of early recurrence of hepatocellular carcinoma. The standard scans for CT liver enhancement in hospitals are four phases. A non-contrast-enhanced (NC) scan was performed prior to the contrast injection. The post-injection phase included the arterial (ART) phase (30–40 s after the contrast injection), the portal vein (PV) phase (70–80 s after the contrast injection), and the delayed (DL) phase (3–5 min after the contrast injection). Since the DL and PV phases provide overlapping information and adding delayed phases not only increases the workload but also adds burden of patients, only the first three phases (i.e., NC, ART and PV) are usually captured and used for diagnosis in many clinical practices (43, 44). We also only use the NC, ART, and PV phases for this study. Our CT images were acquired using two scanners: a GE LightSpeed VCT scanner (GE Medical Systems, Milwaukee, WI, USA) and a Siemens SOMATON Definition AS scanner (Siemens Healthcare, Forchheim, Germany). The resolution of these CT images was 512 ×512, and the thickness of each slice was either 5 or 7 mm. The region of interest (ROI) was manually marked by an abdominal radiologist having 3 years of experience via ITK-SNAP (version 3.6.0, University of Pennsylvania, Philadelphia, USA) (39). It was then corrected by a radiologist having 6 years of experience in the field. In our experiments, we used the physician-labeled ROI as the input to the model. The tumor and liver would behave differently at different stages, which means that the multi-phase CT would show more information. Figure 1 shows the CT images of a patient who underwent a contrast-enhanced scan before surgery.

## Method

**Figure 2:**
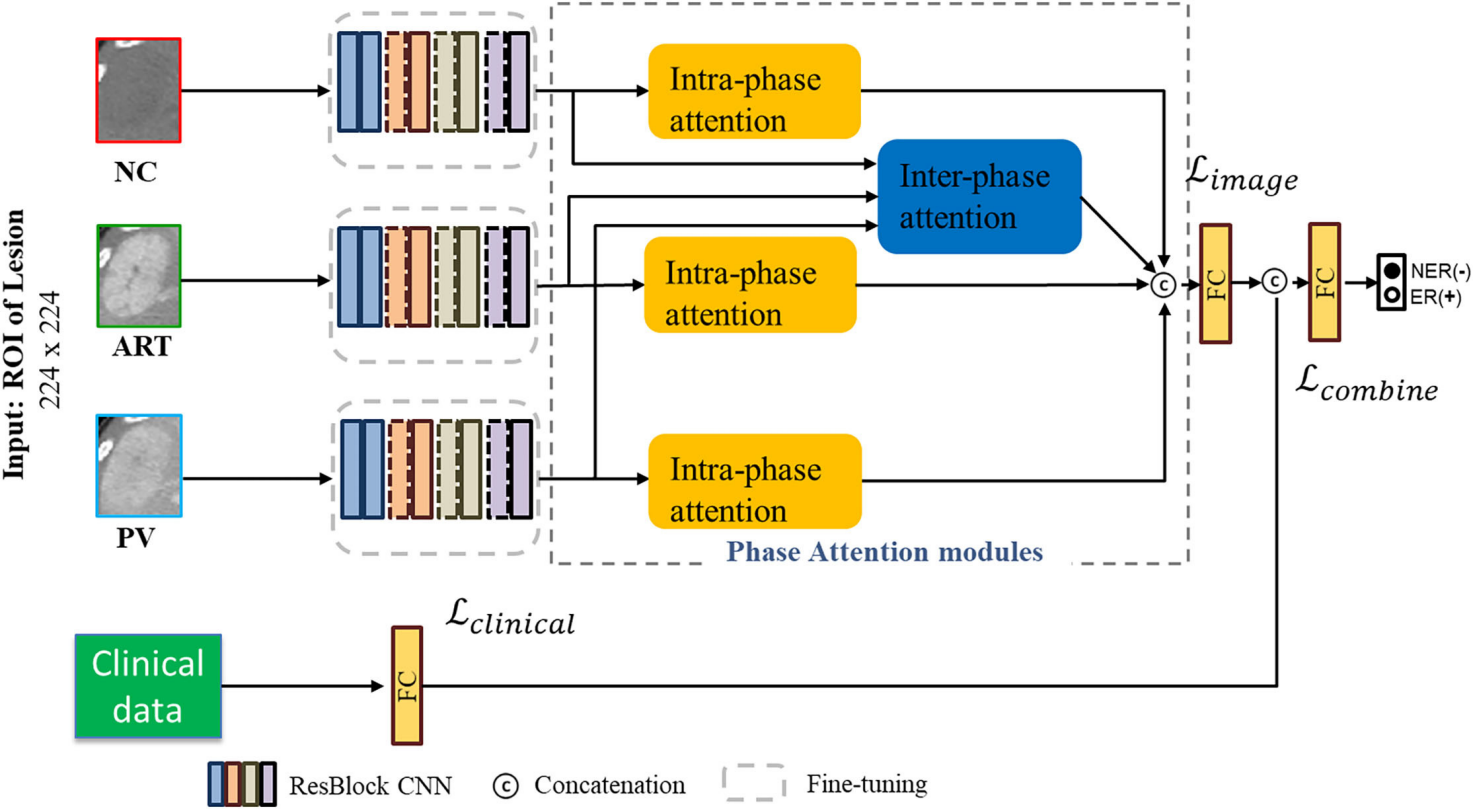
Workflow of the DPA model for joint image data and clinical data. The image pathway consists of three residual CNN blocks and two phase-attention modules. Deep image features are derived from these two components and fed into the fully connected (FC) layer. Clinical data are passed through an FC layer and then concatenated with image features to pass through a final FC layer for early recurrence prediction of HCC.

We propose an end-to-end deep learning prediction model that combines imaging data and clinical information of HCC patients. Figure 2 shows the workflow of our proposed deep phase attention (DPA) model, which directly predicts early recurrence of HCC from multi-phase CT inputs and clinical data. The DPA model is composed of two pathways: image and clinical. The image pathway consists of three residual convolution branches and two phase-attention modules. It is a self-designed prediction network based on the deep residual network (ResNet) backbone (45). In the next subsections, we will introduce our proposed DPA model in detail in terms of the backbone network, the phase attention module, and the clinical data combined with images.

### ResNet Backbone

**Figure 3:**
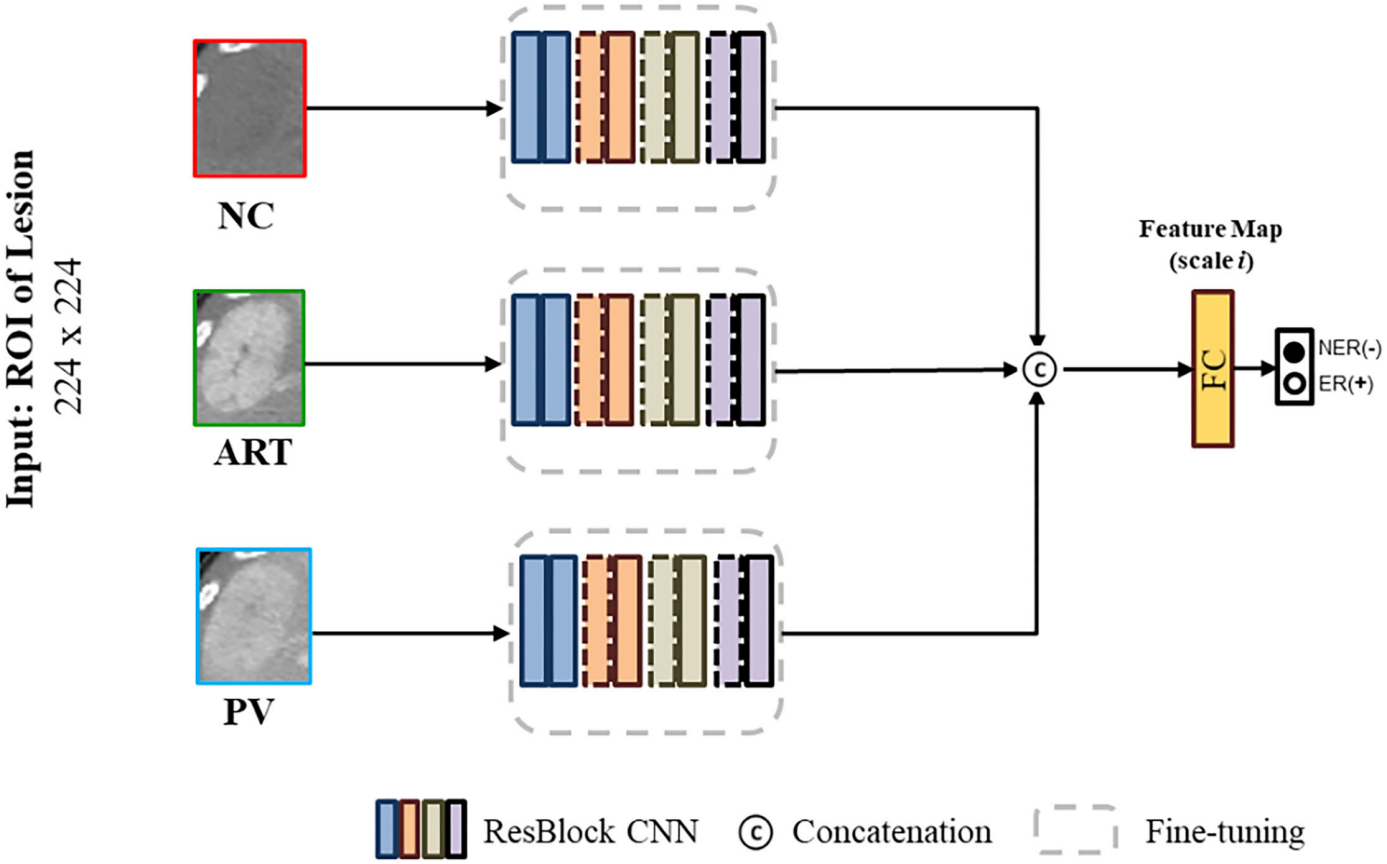
Basic backbone network that was designed based on ResNet.

The proposed deep residual network (ResNet) is a milestone event in the history of CNN images (45). The degradation problem of deep networks indicates that deep networks are not easy to train. Residual blocks can effectively alleviate the network degradation problem and remains a design element of various deep learning networks. We first designed three residual branch CNNs in the same network based on the ResNet structure and extracted high-level features of each of the three phases through these three branches. This backbone network is shown in Figure 3. Our experimental sample size was not large enough; therefore, we used the fine-tuning training method (46), which could alleviate the overfitting problem in network training. We used ImageNet (47) as our pre-training data, and then fine-tuned it using our private data. The detailed residual block design is shown in Table 2.

**Table 2:** ReNet18 residual block structure.

**Layer name**	**Output size**	**ResNet 18** **kernel size, out channels, stride**	**ResNet 50** **kernel size, out channels, stride**
Conv1	112 ×112	7 ×7, 64, stride 2	7 ×7, 64, stride 2
Conv2_x	56 ×56	3 ×3 max pool, stride 2 $\left[\begin{array}{cc} 3\times 3, & 64\\ 3\times 3, & 64 \end{array}\right]\times 2$	3 × 3 max pool, stride 2 $\left[\begin{array}{cc} 1\times 1, & 64\\ 3\times 3, & 64\\ 1\times 1, & 256 \end{array}\right]\times 3\;$
Conv3_x	28 × 28	$\left[\begin{array}{cc} 3\times 3, & 128\\ 3\times 3, & 128 \end{array}\right]\times 2$	$\left[\begin{array}{cc} 1\times 1, & 128\\ 3\times 3, & 128\\ 1\times 1, & 512 \end{array}\right]\times 4$
Conv4_x	14 × 14	$\left[\begin{array}{cc} 3\times 3, & 256\\ 3\times 3, & 256 \end{array}\right]\times 2$	$\left[\begin{array}{cc} 1\times 1, & 256\\ 3\times 3, & 256\\ 1\times 1, & 1024 \end{array}\right]\times 6$
Conv5_x	7 × 7	$\left[\begin{array}{cc} 3\times 3, & 256\\ 3\times 3, & 256 \end{array}\right]\times 2$	$\left[\begin{array}{cc} 1\times 1, & 512\\ 3\times 3, & 512\\ 1\times 1, & 2048 \end{array}\right]\times 3$

### Phase Attention Modules

The phase attention modules were added to extract the important features so that the model could improve the prediction accuracy. At the same time, it did not bring more overheads to the computation and storage of the model. We proposed two types of phase attention modules: intra-phase attention and inter-phase attention.

#### Intra-phase Attention

**Figure 4:**
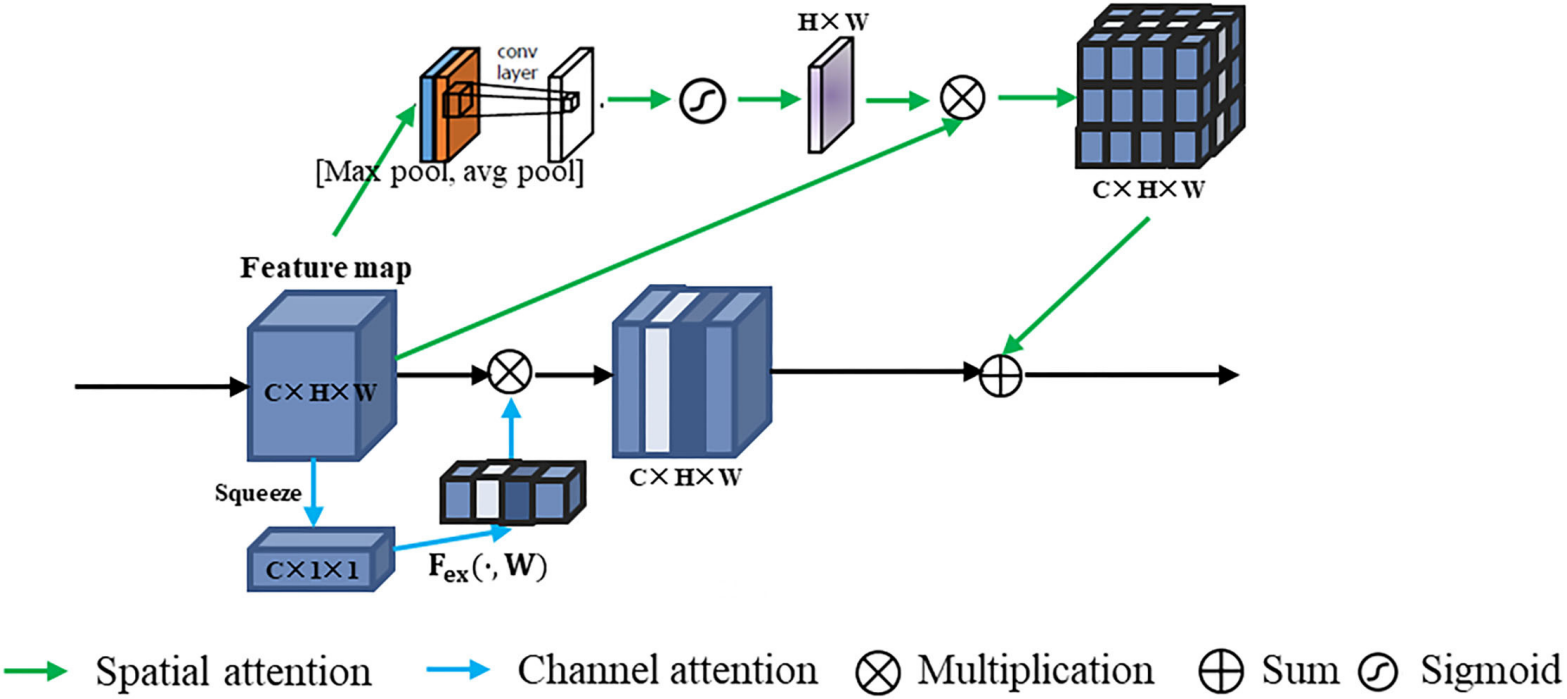
Intra-phase attention structure. All three phase branches are operated in the same way, but we have shown only one phase example here.

We implemented intra-phase attention by using the channel attention and spatial attention modules, namely, squeeze-and-excitation network (SENet) (34) and convolutional block attention module (CBAM) (35). The intra-phase attention module is shown in Figure 4. The intra-phase attention acts independently on each phase. The intra-phase attention module contains channel attention and spatial attention in parallel. For channel attention, a global average pooling (squeeze operation) was first performed on the feature map. By the operation of global pooling (pooling size H × W), we obtained a C × 1 × 1 tensor. Then the excitation operation contains 2 FC layers. The first FC layer has C/r neurons (r is set to 16 in our experiments), which is a dimensionality reduction process. The second FC layer is then up-dimensioned to C neurons, which has the advantage of adding more non-linear processing to fit the complex correlations between channels. Then a sigmod layer is connected to obtain C × 1 × 1 weights. The original feature map (C × H × W) and the C × 1 × 1 attention features are scaled. For spatial attention, a channel-based global max pooling and global average pooling were performed to obtain two H × W feature maps. After that, the two feature maps were concatenated (channel splicing) based on the channel. Then after a 7 × 7 convolution operation, the dimensionality is reduced to 1 channel again. Then, the spatial attention feature was generated by the sigmoid function, and the feature was multiplied by the original feature map. The final feature was obtained by summing up the features generated by the channel attention and spatial attention.

#### Inter-phase Attention

**Figure 5:**
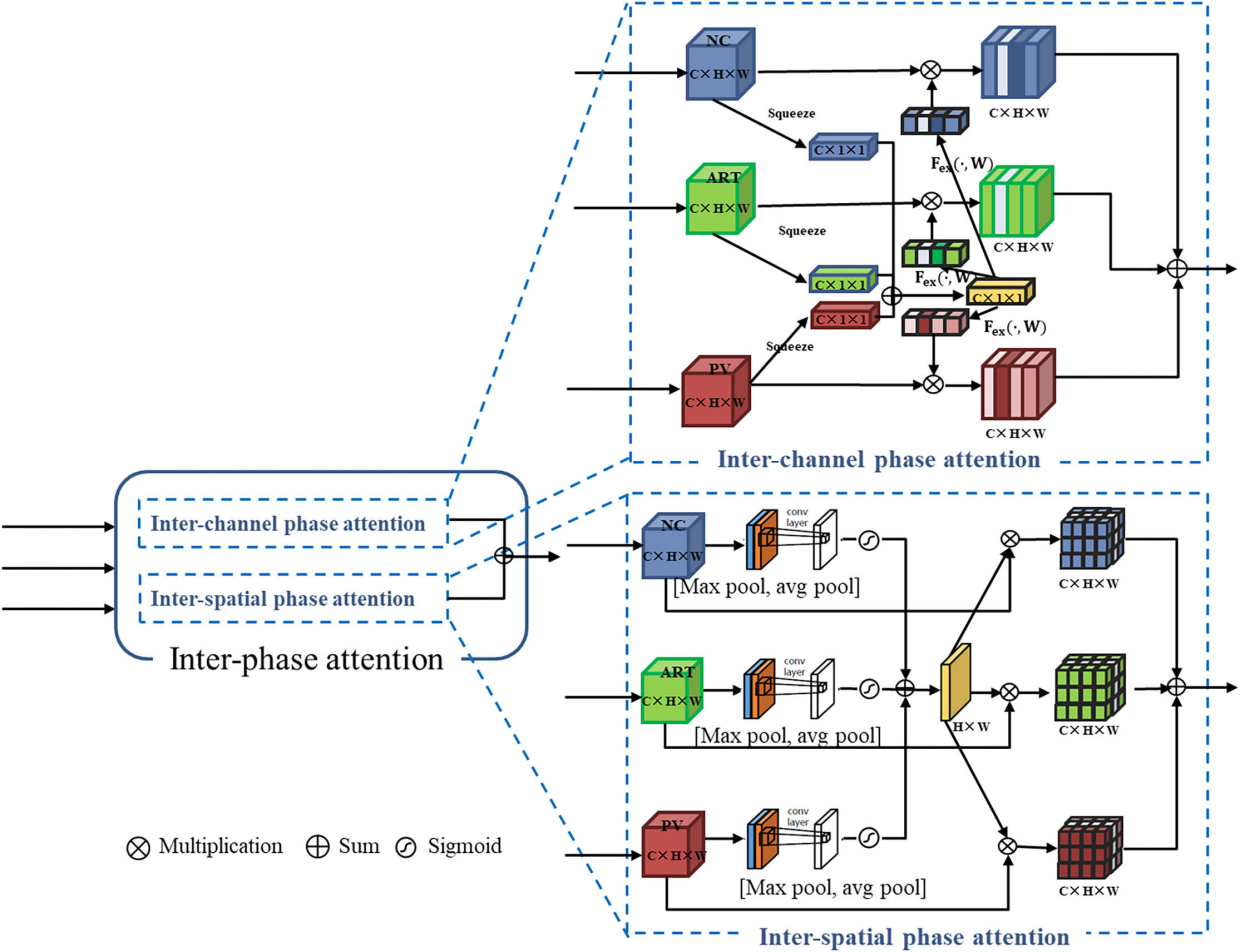
Inter-phase attention structure containing inter-channel phase attention and inter-spatial phase attention: they are in parallel.

In the inter-phase attention module, the feature maps of the three phases are input to the channel attention and spatial attention blocks (see Figure 5). The inter-phase channel attention module differs from the intra-phase channel attention module in the channel attention features (C × 1 × 1). In the inter-phase attention module, the channel features generated by each of the three phases are summed and averaged to generate a new channel attention feature (C × 1 × 1; see the yellow part of the inter-channel phase attention in Figure 5). Then, the scale operation was performed with the original feature maps of each of the three phases. Similarly, the spatial attention features (H × W × 1) generated by each of the three phases were summed and averaged to generate a new spatial attention feature (H × W × 1; see the yellow part of inter-spatial phase attention in Figure 5). Then, the multiplication operation was performed using the original feature maps of the three phases. Finally, all the generated feature maps were summed to produce the output of this inter-spatial attention module.

### Fusion Model Using Clinical Data and CT Images

As shown in Figure 2, our proposed DPA model uses different types of data as the input to the deep learning network. The image features (2,048) were extracted by the image pathway after the CNN module and phase attention module. The clinical data vector had only nine elements; therefore, we added an FC layer to the clinical data pathway to up-dimension it to 30. We also added an FC layer to the image pathway to down-dimension to 30. Then, we concatenated these two types of data features. The early recurrence prediction of HCC was performed by the last FC layer (softmax). We used cross entropy as the loss function of the DPA model. Let *N* be the number of samples. **I**_*j*_ and **c**_*j*_ are the *j*-th CT image input data and clinical input data (*i* = 1, 2, …*N*), respectively. We used *b*, *k*, and ***W*** to denote the bias term, number of neurons, and weight of the last FC layer, respectively; *y*_*j*_ denotes the label. *T*(**c**_*j*_) and *S*(**I**_*j*_) represent the output of the clinical data training pathway and the image training pathway, respectively, before the last FC layer; ⊕ denotes the concatenation operation. The loss function is given as follows:


(1) $$\begin{array}{l} L=CrossEntropy\left(S\left({\text{I}}_{j}\right)⊕T\left({\text{c}}_{j}\right)\right)\\ \;=-\frac{1}{N}\overset{N}{\underset{j=1}{\sum }}\left[y_{j}\ln\;p_{j1}+\left(1-y_{j}\right)\ln\left(p_{j2}\right)\right] \end{array}$$

(2) $$\begin{array}{lll} p_{jk} & = & \;\frac{e^{z_{k}}}{\overset{2}{\underset{k=1}{\sum }}e^{z_{k}}} \end{array}$$

(3) $$\begin{array}{lll} z_{k} & = & W_{k}^{T}\cdot \left[S\left({\text{I}}_{j}\right)⊕T\left(c_{j}\right)\right]+b_{k}\;,\;k=1,2. \end{array}$$

#### Joint Loss Function

In the DPA model, we added a softmax layer to the clinical data pathway and CT image pathway before concatenation. With this, we could calculate the loss of both pathways. Let $p_{j1}^{I}$ represent the output possibility of the image pathway; $p_{j1}^{c}\;$represent the output possibility of the clinical data pathway; and $p_{j1}^{com}\;$represent the output possibility of the concatenate pathway. Then, the joint loss is given as follows:


(4) $$\begin{array}{l} L=\frac{1}{4}L_{image}+\frac{1}{4}L_{clinical}+\frac{1}{2}L_{combine}=\frac{1}{4}CrossEntropy\left(S\left({\text{I}}_{j}\right)\right)\\ +\frac{1}{4}CrossEntropy\left(T\left({\text{I}}_{j}\right)\right)+\frac{1}{2}CrossEntropy\left(S\left({\text{I}}_{j}\right)⊕T\left({\text{c}}_{j}\right)\right) \end{array}$$

where $L_{image}\;$represents the loss of the image pathway; $L_{clinical}\;$represents the loss of the clinical data pathway; and $L_{combine}\;$represents the loss after combination. $L_{combine}$ is calculate by the loss function (Equation 1).

### Experiments

Our experiments were based on the Python 3.6 language environment. We implemented our network using the Tensorflow and Keras frameworks. All our experiments were conducted on a machine with the following specifications: Intel(R) Core(TM) i7-8700K CPU @ 3.70GHz 64-bit, RAM 32G, GPU NVIDIA GeForce GTX 1080 Ti, Windows 10 Professional version number: 19042.1415.

We used ten-fold cross-validation as our evaluation method. The accuracy and the area under the curve (AUC) for the receiver operating characteristics were calculated to evaluate the prediction performance of the model. We randomly divided 167 patients into 10 groups; each group contained 6 or 7 ERs and 10 or 11 NERs. During the ten-fold cross-validation, we selected one data group as the test group, and the remaining nine groups were training groups. The mean value was calculated for the results obtained from the ten sets of experiments. This mean value was used as the final score of the model. The number of CT slices containing the tumor varied from patient to patient because of the different tumor sizes and locations. We selected the central slice (the one with the largest tumor cross-section) as well as its adjacent slices as our data set. A total of 765 labeled slices were used in our experiments. Table 3 summarizes the number of training images and test images (CT slice images) for each experiment.

**Table 3:** The distribution of the 10-fold cross-validation dataset.

**Experiment**	**E1**	**E2**	**E3**	**E4**	**E5**	**E6**	**E7**	**E8**	**E9**	**E10**
Training	695 (150)	681 (150)	683 (150)	691 (150)	694 (150)	676 (150)	700 (150)	694 (151)	680 (151)	691 (151)
Testing	70 (17)	84 (17)	82 (17)	74 (17)	71 (17)	89 (17)	65 (17)	71 (16)	85 (16)	74 (16)
Total	765 (167)	765 (167)	765 (167)	765 (167)	765 (167)	765 (167)	765 (167)	765 (167)	765 (167)	765 (167)

*E1~10 is 10 sets of experiments.*

*Outside the brackets is the number of slices. The bracket indicates the number of cases (patients)*.

We used the following parameters in the training process: a batch size of 8, a default training epoch of 50, a learning rate of 0.0001 for finetuning training, and a loss function as described in Section Fusion Model Using Clinical Data and CT Images. In the model with the addition of joint loss, the training parameters were the same, and the loss function is described in Equation (4).

#### Comparison Results of Different Models

We first compared the prediction results of ReNet18 and ResNet50 as the residual branching blocks. From Table 4, we can see that the performance of ResNet18 is almost the same as that of ResNet50, but ResNet18 has fewer network layers and parameters and a lower training time. Therefore, we chose the residual block of ResNet18 as our backbone CNN.

**Table 4:** Prediction results of ReNet18 and ResNet50 based branch CNN.

**Model**	**ResNet50 based (pretrain)**		**ResNet18 based (pretrain)**	
	**Accuracy**	**AUC**	**Accuracy**	**AUC**
Average	62.5% ± 5.5	0.662 ± 0.08	62.3% ± 6.0	0.662 ± 0.04

*Acc, accuracy; AUC, area under the receiver operating characteristics curve*.

We compared the proposed method with different existing models in Table 5. The existing models include the clinical model (32), the radiomics model (48), deep learning models (32, 33) and deep attention models (34, 35). The clinical model is a random forest approach that uses only clinical data to construct a random forest, as described in (32). To extract radiomic features and select features using LASSO, we used the radiomics model given in (48). Finally, a random forest was used to build the prediction model. Also, we compared the proposed method with our previous deep learning-based work (32, 33) and the deep attention models of SENet (34) and CBAM (35). In our previous study (32), we input three phases as three channels into a single network, which we considered as early fusion. The original SENet and CBAM were not applicable to multi-period phase input data. Here, we applied the attention mechanisms of SENet and CBAM to the three-branched backbone network of ResNet18. The comparative results of the experiments are shown in Table 5. Our experiment results prove that the clinical model predicts better than the imaging model (radiomics model or deep learning model) with our batch of data. Among the imaging models, our proposed DPA model performs the best. Our proposed DPA fusion model, which combined multi-phase CT and clinical data, achieved a prediction accuracy of 81.2% and an AUC of 0.869; our proposed model outperformed other models.

**Table 5:** Comparison results of different models.

**Input data type**	**Models**	**Acc**	**AUC**
Clinical data only	Clinical model (32)	76.03% ± 10.0	0.753 ± 0.13
Image data only	Radiomics model (48)	67.04% ± 4.9	0.640 ± 0.03
	WANG et al.'s Model (32)	69.5% ± 5.1	0.723 ± 0.06
	SENet-like model (34)	64.5% ± 3.4	0.675 ± 0.06
	CBAM-like model (35)	66.7% ± 4.2	0.684 ± 0.05
	Propose DPA model	70.7% ± 2.4	0.747 ± 0.03
Clinical data and Image data	WANG et al.'s fusion model (33)	80.49% ± 4.3	0.833 ± 0.03
	Propose DPA fusion model	**81.2%** **±1.3**	**0.869** **±0.03**

*Acc, accuracy; AUC, area under the receiver operating characteristics curve. The bold values indicate P values < 0.05 are highlighted. The bold values indicate the best results*.

#### Ablation Study

To demonstrate the effectiveness of intra-phase attention and inter-phase attention in the DPA model, we compared the fusion strategies of the proposed phase attention module. We used the three-branch ResNet18 as the network backbone of all fusion strategies. The comparison results are shown in Table 6. Note that all the methods using phase attention outperformed the network without phase attention, which proves the effectiveness of phase attention. Moreover, the experiments proved that more improvements are obtained by adding both intra-phase attention and inter-phase attention. The inclusion of clinical data and joint loss strategy are also ways to improve the prediction performance. In particular, the DPA fusion model can better predict early recurrence in HCC patients after adding clinical data.

**Table 6:** Prediction results by ablation study.

	**Intra-phase**	**Inter-phase**	**Clinical data**	**Joint loss**	**Acc**	**AUC**
Baseline					62.3% ± 6.0	0.662 ± 0.04
Model1	√				67.2% ± 3.1	0.692 ± 0.04
Model2		√			67.6% ± 2.5	0.695 ± 0.04
Model3	√	√			70.7% ± 2.4	0.747 ± 0.03
Model4	√	√	√		80.5% ± 2.5	0.849 ± 0.04
Model5 (proposed)	√	√	√	√	**81.2%** **±1.3**	**0.869** **±0.03**

*The best results are highlighted in bold*.

## Discussion

Contrast-enhanced CT is one of the most important modalities for liver tumor diagnosis. Multi-phase CT images provide rich and complementary information for the diagnosis of liver tumors. Based on clinical observations, the PV phase is the preferred choice for liver tumor segmentation. In our experiments with ResNet18 and using only single-phase CT images, the highest prediction accuracy was achieved for the PV phase, as shown in Table 7. This is consistent with the clinical observation that the PV phase provides doctors with clearer information, such as contours. In this study, we propose a multi-branch ResNet18 backbone model using multi-phase images as multiple inputs to improve the early recurrence prediction performance of HCC using information from multiple phases. Our method improved the prediction accuracy by at least 8% in both cases as compared with the network using only single-phase images; this demonstrated the effectiveness of the multi-branch ResNet18 backbone model and the efficient use of multi-phase CT images.

**Table 7:** Comparison of single-phase ResNet18 backbone networks.

**Model**	**ResNet18 (NC)**		**ResNet18 (ART)**		**ResNet18 (PV)**	
	**Acc**	**AUC**	**Acc**	**AUC**	**Acc**	**AUC**
Average	58.7% ± 6.4	0.627 ± 0.09	60.3% ± 4.1	63.24 ± 0.06	**61.42** ± 6.6	**65.76** **±0.06**

*The best results are highlighted in bold*.

We demonstrated the effectiveness of the phase attention module through an ablation study (see Table 6). When intra-phase and inter-phase attention were used together, the accuracy of the backbone network improved by 8.4%. In contrast to the SENet-like and CBAM-like systems, our proposed attention mechanism was designed with an additional inter-phase attention module. Natural images do not use multi-phase inter-attention mechanisms, but in the medical field, multi-phase medical images are commonly available, such as multi-phase CT and multi-phase MR. These images are not only applicable to the study of liver tumors but can also be applied to the analysis of other organ diseases. In the future, we will apply the DPA model to other topics as a method to further validate the effectiveness and scalability of the phase attention module.

Our study has certain limitations mainly because we implemented a deep learning approach on a small sample dataset. To avoid overfitting, we expanded the training sample by data augmentation and used fine-tuning for training. The experiment results look good, but deep learning training requires more training data. Moreover, in our previous data collection, the DL phase of the patients was missing, which prevented the DL phase from being included in the network training. Although the DPA model based on three-phase CT images performed well in the predictions, the performance can be further improved by using the information of the DL phase. In our future work, we will collect more extensive data. We propose that deep learning models can extract high-level features. However, the high-level radiological features extracted by the convolutional layers may suffer from low medical interpretability and high overfitting probability, especially when the training dataset is not large enough for understanding and making diagnostic decisions. In the future, we will consider more image features, such as incorporating histology-extracted features into the deep learning network; these measures will increase medical interpretability and make the model better for use in clinical practice.

## Conclusion

The performance of the deep learning model was improved by adding intra-phase attention and inter-phase attention. Our proposed fusion model, which combined multi-phase CT and clinical data, achieved a prediction accuracy of 81.2% and an AUC of 0.869.

## Data Availability Statement

The data analyzed in this study is subject to the following licenses/restrictions: the data that support the findings of this study are available on request from the corresponding author. The data are not publicly available due to privacy or ethical restrictions. Requests to access these datasets should be directed to chen@is.ritsumei.ac.jp, llf@zju.edu.cn, and hongjiehu@zju.edu.cn.

## Ethics Statement

The studies involving human participants were reviewed and approved by Ritsumeikan University, Zhejiang University, and Run Run Shaw Hospital. Written informed consent for participation was not required for this study in accordance with the national legislation and the institutional requirements.

## Author Contributions

WW: contributed to conception and design of the study and wrote the first draft of the manuscript. FW and QC: performed the statistical analysis and data collection. SO: performs network training and tunes parameters and provides ideas. YI, XH, LL, and Y-WC: manuscript writing, image acquisition, editing, and reviewing. RT and HH: idea generation and reviewing. All authors contributed to manuscript revision, read, and approved the submitted version.

## Funding

This work was supported in part by the Grant in Aid for Scientific Research from the Japanese Ministry for Education, Science, Culture and Sports (MEXT) under the Grant Nos. 20KK0234, 21H03470, and 20K21821, and in part by the Natural Science Foundation of Zhejiang Province (LZ22F020012), in part by Major Scientific Research Project of Zhejiang Lab (2020ND8AD01), and in part by the National Natural Science Foundation of China (82071988), the Key Research and Development Program of Zhejiang Province (2019C03064), the Program Co-sponsored by Province and Ministry (No. WKJ-ZJ-1926) and the Special Fund for Basic Scientific Research Business Expenses of Zhejiang University (No. 2021FZZX003-02-17).

## Conflict of Interest

The authors declare that the research was conducted in the absence of any commercial or financial relationships that could be construed as a potential conflict of interest.

## Publisher's Note

All claims expressed in this article are solely those of the authors and do not necessarily represent those of their affiliated organizations, or those of the publisher, the editors and the reviewers. Any product that may be evaluated in this article, or claim that may be made by its manufacturer, is not guaranteed or endorsed by the publisher.

## References

[1] Elsayes KM Kielar AZ Agrons MM Szklaruk J Tang A Bashir MR . Liver imaging reporting and data system: an expert consensus statement. J Hepatocel Carcinoma. (2017) 4:29–39. 10.2147/JHC.S125396 28255543 PMC5322844

[2] Zhu RX Seto WK Lai CL. Epidemiology of hepatocellular carcinoma in the Asia-Pacific region. Gut Liver. (2016) 10:332–9. 10.5009/gnl15257 27114433 PMC4849684

[3] Thomas MB Zhu AX. Hepatocellular carcinoma: the need for progress. J Clin Oncol. (2005) 23:2892–9. 10.1200/JCO.2005.03.196 15860847

[4] Yang T Lin C Zhai J Shi S Zhu M Zhu N . Surgical resection for advanced hepatocellular carcinoma according to Barcelona Clinic Liver Cancer (BCLC) staging. J Cancer Res Clin Oncol. (2012) 138:1121–9. 10.1007/s00432-012-1188-0 22402598 PMC11824283

[5] Portolani N Coniglio A Ghidoni S Giovanelli M Benetti A Tiberio GAM . Early and late recurrence after liver resection for hepatocellular carcinoma: prognostic and therapeutic implications. Ann Surg. (2006) 243:229–35. 10.1097/01.sla.0000197706.21803.a1 16432356 PMC1448919

[6] Shah SA Cleary SP Wei AC Yang I Taylor BR Hemming AW . Recurrence after liver resection for hepatocellular carcinoma: risk factors, treatment, and outcomes. Surgery. (2007) 141:330–9. 10.1016/j.surg.2006.06.028 17349844

[7] Feng J Chen J Zhu R Yu L Zhang Y Feng D . Prediction of early recurrence of hepatocellular carcinoma within the Milan criteria after radical resection. Oncotarget. (2017) 8:63299–310. 10.18632/oncotarget.18799 28968990 PMC5609922

[8] Cheng Z Yang P Qu S Zhou J Yang J Yang X . Risk factors and management for early and late intrahepatic recurrence of solitary hepatocellular carcinoma after curative resection. HPB. (2015) 17:422–7. 10.1111/hpb.12367 25421805 PMC4402053

[9] Liu J Zhu Q Li Y Qiao G Xu C Guo D . Microvascular invasion and positive HB e antigen are associated with poorer survival after hepatectomy of early hepatocellular carcinoma: a retrospective cohort study. Clin Res Hepatol Gastroenterol. (2018) 42:330–8. 10.1016/j.clinre.2018.02.003 29551612

[10] Qiao W Yu F Wu L Li B Zhou Y. Surgical outcomes of hepatocellular carcinoma with biliary tumor thrombus: a systematic review. BMC Gastroenterol. (2016) 16:1–7. 10.1186/s12876-016-0427-2 26822229 PMC4730620

[11] Guerrini GP Pinelli D Benedetto FD Marini E Corno V Guizzetti M . Predictive value of nodule size and differentiation in HCC recurrence after liver transplantation. Surg Oncol. (2016) 25:419–28. 10.1016/j.suronc.2015.09.003 26403621

[12] Ho WH Lee KT Chen HY Ho TW Chiu HC. Disease-free survival after hepatic resection in hepatocellular carcinoma patients: a prediction approach using artificial neural network. PLoS ONE. (2012) 7:e29179. 10.1371/journal.pone.0029179 22235270 PMC3250424

[13] Shim JH Jun MJ Han S Lee YJ Lee SG Kim KM . Prognostic nomograms for prediction of recurrence and survival after curative liver resection for hepatocellular carcinoma. Ann Surg. (2015) 261:939–46. 10.1097/SLA.0000000000000747 24950276

[14] Hirokawa F Hayashi M Miyamoto Y Asakuma M Shimizu T Komeda K . Outcomes and predictors of microvascular invasion of solitary hepatocellular carcinoma. Hepatol Res. (2014) 44:846–53. 10.1111/hepr.12196 23834279

[15] Sterling RK Wright EC Morgan TR Seeff LB Hoefs JC Bisceglie AMD . Frequency of elevated hepatocellular carcinoma (HCC) biomarkers in patients with advanced hepatitis C. Am J Gastroenterol. (2012) 107:64. 10.1038/ajg.2011.312 21931376 PMC3903319

[16] Lambin P Rios-Velazquez E Leijenaar R Carvalho S van Stiphout RGPM Granton P . Radiomics: extracting more information from medical images using advanced feature analysis. Eur J Cancer. (2012) 48:441–446. 10.1016/j.ejca.2011.11.036 22257792 PMC4533986

[17] Coppola F Faggioni L Gabelloni M Vietro FD Mendola V Cattabriga A . Human, all too human? An all-around appraisal of the AI revolution in medical imaging. Front Psychol. (2021) 12:710982. 10.3389/fpsyg.2021.710982 34650476 PMC8505993

[18] Gillies RJ Kinahan PE Hricak H. Radiomics: images are more than pictures, they are data. Radiology. (2015) 278:563–77. 10.1148/radiol.2015151169 26579733 PMC4734157

[19] Braman NM Etesami M Prasanna P Dubchuk C Gilmore H Tiwari P . Intratumoral and peritumoral radiomics for the pretreatment prediction of pathological complete response to neoadjuvant chemotherapy based on breast DCE-MRI. Breast Cancer Res. (2017) 19:57. 10.1186/s13058-017-0846-1 28521821 PMC5437672

[20] Ma X Wei J Gu D Zhu Y Feng B Liang M . Preoperative radiomics nomogram for microvascular invasion prediction in hepatocellular carcinoma using contrast-enhanced CT. Eur Radiol. (2019) 29:3595–605. 10.1007/s00330-018-5985-y 30770969

[21] Zhou Y He L Huang Y Chen S Wu P Ye W . CT-based radiomics signature: a potential biomarker for preoperative prediction of early recurrence in hepatocellular carcinoma. Abdom Radiol. (2017) 42:1695–704. 10.1007/s00261-017-1072-0 28180924

[22] Ning P Gao F Hai J Wu M Chen J Zhu S . Application of CT radiomics in prediction of early recurrence in hepatocellular carcinoma. Abdom Radiol. (2019) 45:64–72. 10.1007/s00261-019-02198-7 31486869

[23] Hosny A Parmar C Quackenbush J Schwartz LH Aerts HJWL. Artificial intelligence in radiology. Nat Rev Cancer. (2018) 18:500–510. 10.1038/s41568-018-0016-5 29777175 PMC6268174

[24] Scapicchio C Gabelloni M Barucci A Cioni D Saba L Neri E. A deep look into radiomics. La radiologia medica. (2021) 126:1296–311. 10.1007/s11547-021-01389-x 34213702 PMC8520512

[25] Coppola F Giannini V Gabelloni M Panic J Defeudis A Monaco SL . Radiomics and magnetic resonance imaging of rectal cancer: from engineering to clinical practice. Diagnostics. (2021) 11:756. 10.3390/diagnostics11050756 33922483 PMC8146913

[26] Gabelloni M Faggioni L Borgheresi R Restante G Shortrede J Tumminello L . Bridging gaps between images and data: a systematic update on imaging biobanks. Eur Radiol. (2022). 10.1007/s00330-021-08431-6. [Epub ahead of print]. 35001159

[27] Afshar P Mohammadi A Plataniotis KN Oikonomou A Benali H. From handcrafted to deep-learning-based cancer radiomics: challenges and opportunities. IEEE Sig Proces Mag. (2019) 36:132–60. 10.1109/MSP.2019.2900993

[28] Peng H Dong D Fang MJ Li L Tang LL Chen L . Prognostic value of deep learning PET/CT-based radiomics: potential role for future individual induction chemotherapy in advanced nasopharyngeal carcinoma. Clin Cancer Res. (2019) 25:4271–9. 10.1158/1078-0432.CCR-18-3065 30975664

[29] Jing B Deng Y Zhang T Hou D Li B Qiang M . Deep learning for risk prediction in patients with nasopharyngeal carcinoma using multi-parametric MRIs. Comput Methods Prog Biomed. (2020) 197:105684. 10.1016/j.cmpb.2020.105684 32781421

[30] Peng Y Bi L Guo Y Feng D Fulham M Kim J. Deep multi-modality collaborative learning for distant metastases predication in PET-CT soft-tissue sarcoma studies. In: 41st Annual International Conference of the IEEE Engineering in Medicine and Biology Society (EMBC). IEEE (2019). p. 3658–88. 10.1109/EMBC.2019.8857666 31946670

[31] Yamashita R Long J Saleem A Rubin DL Shen J. Deep learning predicts postsurgical recurrence of hepatocellular carcinoma from digital histopathologic images. Sci Rep. (2021) 11:1–14. 10.1038/s41598-021-81506-y 33479370 PMC7820423

[32] Wang W Chen Q Iwamoto Y Han X Zhang Q Hu H . Deep learning-based radiomics models for early recurrence prediction of hepatocellular carcinoma with multi-phase CT images and clinical data. In: 41st Annual International Conference of the IEEE Engineering in Medicine and Biology Society (EMBC). IEEE (2019). p. 4881–4. 10.1109/EMBC.2019.8856356 31946954

[33] Wang W Chen Q Iwamoto Y Aonpong P Lin L Hu H . Deep fusion models of multi-phase CT and selected clinical data for preoperative prediction of early recurrence in hepatocellular carcinoma. IEEE Access. (2020) 8:139212–9220. 10.1109/ACCESS.2020.3011145

[34] Hu J Shen L Sun G. Squeeze-and-excitation networks. In: Proceedings of the IEEE Conference on Computer Vision and Pattern Recognition. (2018). p. 7132–41. 10.1109/CVPR.2018.00745

[35] Woo S Park J Lee JY Kweon IS. Cbam: Convolutional block attention module. In: Proceedings of the European conference on computer vision (ECCV). (2018). p. 3–19.

[36] Zamir SW Arora A Khan S Hayat M Khan FS Yang MH . Learning enriched features for real image restoration and enhancement. In: Computer Vision–ECCV 2020: 16th European Conference, Glasgow, UK. Proceedings, Part XXV 16. Springer International Publishing (2020). p. 492–511. 10.1007/978-3-030-58595-2_30

[37] Ibrahim S Roychowdhury A Hean TK. Risk factors for intrahepatic recurrence after hepatectomy for hepatocellular carcinoma. Am J Surg. (2007) 194:17–22. 10.1016/j.amjsurg.2006.06.051 17560903

[38] Chang PE Ong WC Lui HF Tan CK. Is the prognosis of young patients with hepatocellular carcinoma poorer than the prognosis of older patients? A comparative analysis of clinical characteristics, prognostic features, and survival outcome. J Gastroenterol. (2008) 43:881–8. 10.1007/s00535-008-2238-x 19012042

[39] Okamura Y Ashida R Ito T Sugiura T Mori K Uesaka K. Preoperative neutrophil to lymphocyte ratio and prognostic nutritional index predict overall survival after hepatectomy for hepatocellular carcinoma. World J Surg. (2015) 39:1501–9. 10.1007/s00268-015-2982-z 25670038

[40] Yang HJ Guo Z Yang YT Jiang JH Qi YP Li JJ . Blood neutrophil-lymphocyte ratio predicts survival after hepatectomy for hepatocellular carcinoma: a propensity score-based analysis. World J Gastroenterol. (2016) 22:5088–95. 10.3748/wjg.v22.i21.5088 27275101 PMC4886384

[41] Lancaster HO. The Chi-squared Distribution. New York, NY: Wiley (1969).

[42] McHugh ML. The Chi-square test of independence. Biochem Med. (2013) 23:143–9. 10.11613/BM.2013.018 23894860 PMC3900058

[43] Yang Y Zhou Y Zhou C Ma X. Deep learning radiomics based on contrast enhanced computed tomography predicts microvascular invasion and survival outcome in early stage hepatocellular carcinoma. Eur J Surg Oncol. (2021). 10.1016/j.ejso.2021.11.120. [Epub ahead of print]. 34862094

[44] Lee I Huang J Chen T Yen C Chiu N Hwang H . Evolutionary learning-derived clinical-radiomic models for predicting early recurrence of hepatocellular carcinoma after resection. Liver Cancer. (2021) 10:572–82. 10.1159/000518728 34950180 PMC8647074

[45] He K Zhang X Ren S Sun J. Deep residual learning for image recognition. In: Proceedings of the IEEE Conference on Computer Vision and Pattern Recognition (CVPR). (2016). p. 770–8.

[46] Morid MA Borjali A Del Fiol G. A scoping review of transfer learning research on medical image analysis using ImageNet. Comput Biol Med. (2021) 128:104115. 10.1016/j.compbiomed.2020.104115 33227578

[47] Deng J Dong W Socher R Li LJ Li K Li FF. Imagenet: a large-scale hierarchical image database. In: IEEE Conference on Computer Vision and Pattern Recognition (CVPR). IEEE (2009). p. 248–55. 10.1109/CVPR.2009.5206848

[48] Aonpong P Chen Q Iwamoto Y Lin L Hu H Zhang Q . Comparison of machine learning–based radiomics models for early recurrence prediction of hepatocellular carcinoma. J Image Grap. (2019) 7:117–25. 10.18178/joig.7.4.117-125

